# Supplementary material for: FERN – a Java framework for stochastic simulation and evaluation of reaction networks
Source: BMC Bioinformatics. 2008 Aug 29;9:356. doi: 10.1186/1471-2105-9-356 (PMC2553347; doi:10.1186/1471-2105-9-356)
Supplement: Additional file 1 — FERN distribution, Version 1.3. This archive contains the FERN source code and binaries as well as documentation and example models in FernML and SBML. [file 1471-2105-9-356-S1.zip › fern/doc/javadoc/fern/network/sbml/package-use.html]

Uses of Package fern.network.sbml


---


|  |  |  |
| --- | --- | --- |
| **Overview** \| **Package** \| Class \| **Use** \| **Tree** \| **Deprecated** \| **Index** \| **Help** | |  |
| PREV   NEXT | **FRAMES**    **NO FRAMES**     **All Classes** |  |


---


## **Uses of Package fern.network.sbml**

| Packages that use fern.network.sbml | |
| --- | --- |
| **fern.cellDesigner** |  |
| **fern.network.sbml** | Provides the classes for parsing and using sbml based networks. |
| **fern.tools** | Provides common used tools. |

Classes in fern.network.sbml used by fern.cellDesigner

****SBMLPropensityCalculator****
  
          Propensity calculator which is used for `SBMLNetwork`s.

Classes in fern.network.sbml used by fern.network.sbml

****MathTree****
  
          Representation of am evaluation tree.

****MathTree.Node****

****SBMLNetwork****
  
          For specifications of the sbml format refer to http:\\www.sbml.org.

Classes in fern.network.sbml used by fern.tools

****MathTree****
  
          Representation of am evaluation tree.

---


|  |  |  |
| --- | --- | --- |
| **Overview** \| **Package** \| Class \| **Use** \| **Tree** \| **Deprecated** \| **Index** \| **Help** | |  |
| PREV   NEXT | **FRAMES**    **NO FRAMES**     **All Classes** |  |


---
